# Homozygosity mapping and sequencing identify two genes that might contribute to pointing behavior in hunting dogs

**DOI:** 10.1186/s40575-015-0018-5

**Published:** 2015-04-18

**Authors:** Denis A Akkad, Wanda M Gerding, Robin B Gasser, Jörg T Epplen

**Affiliations:** Human Genetics, Ruhr University Bochum, Universitaetsstrasse 150, Bochum, 44801 Germany; Faculty of Veterinary and Agricultural Sciences, The University of Melbourne, Parkville, Victoria 3010 Australia; Faculty of Health, University Witten-Herdecke, Alfred-Herrhausen-Strasse 50, Witten, 58448 Germany

**Keywords:** Dog, Gene chip, NGS, Pointing, Behavior

## Abstract

**Background:**

The domestic dog represents an important model for studying the genetics of behavior. In spite of technological advances in genomics and phenomics, the genetic basis of most specific canine behaviors is largely unknown. Some breeds of hunting dogs exhibit a behavioral trait called “pointing” (a prolonged halt of movement to indicate the position of a game animal). Here, the genomes of pointing dogs (Large Munsterlander and Weimaraner) were compared with those of behaviorally distinct herding dogs (Berger des Pyrenées and Schapendoes). We assumed (i) that these four dog breeds initially represented inbred populations and (ii) that selective breeding for pointing behavior promotes an enrichment of the genetic trait in a homozygous state.

**Results:**

The homozygosity mapping of 52 dogs (13 of each of the four breeds) followed by subsequent interval resequencing identified fixed genetic differences on chromosome 22 between pointers and herding dogs. In addition, we identified one non-synonomous variation in each of the coding genes *SETDB2* and *CYSLTR2* that might have a functional consequence. Genetic analysis of additional hunting and non-hunting dogs revealed consistent homozygosity for these two variations in six of seven pointing breeds.

**Conclusions:**

Based on the present findings, we propose that, together with other genetic, training and/or environmental factors, the nucleotide and associated amino acid variations identified in genes *SETDB2* and *CYSLTR2* contribute to pointing behavior.

**Electronic supplementary material:**

The online version of this article (doi:10.1186/s40575-015-0018-5) contains supplementary material, which is available to authorized users.

## Lay summary

### Variations in two genes may contribute to pointing behavior in hunting dogs

Man’s best friend, the dog, has evolved through domestication and breeding thus promoting different traits in specific breeds. Hunting dogs are bred to hunt with or for humans, and in some of the breeds the so called pointing behavior is fixed. Thereby the dog stops moving and directs to game with the muzzle. In order to investigate the genetic basis of this behavioral trait, two pointing breeds (Large Muensterlaender, Weimaraner) were compared to herding breeds that do not point (Berger des Pyrenees, Schapendoes). Searching for genes only present in pointing dogs but not in sheep dogs, a genomic region was identified on chromosome 22 in pointers. This very region was also verified in other pointing dog breeds, but not in breeds that do not point. Moreover, the region includes genomic pecularities potentially resulting also in physiological alterations. Thus the two genes identified may, together with other genetic and environmental factors, contribute to pointing behavior.

## Background

The behavior of dogs and their interactions with humans and/or other species are fascinating and the subject of intense research [1]. Although it has been challenging to associate dog behavior with genotype [2], some researchers have hypothesized that some behavioral (phenotypic) traits that are tightly linked to particular breeds might be genetically programmed [3,4].

Pointing is a behavioral trait exhibited by many carnivores [5] where the pointing pause allows the predator to locate prey accurately using scent or sound, in preparation for a pounce. Some but not all hunting dogs have this trait, which usually represents a prolonged halt of movement prior to a pounce, in which the dog indicates the position of game to its master or an accompanying hunter [6]. A review of the literature indicates that the characteristic stop-before-the-pounce behavior has been accentuated through selective breeding, leading to the pronounced and pointing behavior of today’s dogs. Pointing in hunting dogs was refined over centuries and apparently had two different origins. Early uses of hunting dogs in both Europe [7] and North America [8] included dogs trailing large game animals by scent or chasing them by sight. There is evidence that pointing and other hunting traits (such as searching and tracking) are heritable in the breeds Large Munsterlander and German Shorthaired Pointing dogs [9]. In contrast to pointing dogs, the herding dogs, such as Berger des Pyrenées, Border Collies and Schapendoes, have an innate ability or trait to control the movement of other animals, such as sheep or cattle [10]. Interestingly, explicit pointing behavior is usually absent from the latter breeds [9], and herding ability is usually absent from breeds that point [9]. In spite of the pronounced behavioral differences between these two groups of dogs, nothing is known about the molecular or genetic basis of each of these traits.

The availability of the dog genome sequence [11] and advanced DNA methods, such as microarray and high throughput sequencing [12,13], provides unique opportunities to explore, for the first time in detail, the genetic constitution of individual dogs and to undertake comparative genomic analyses of different breeds of dogs (on a population scale), to suggest links between behavioral (or other) phenotypes and genotypes. Given our specific interest in the behavioral differences between pointing and herding dogs, we asked the question as to whether pointing behavior in particular breeds, such as the Large Munsterlander and Weimaraner, can be explained by a contribution of one or more gene loci in a homozygous state by comparison with herding and/or other dogs that do not exhibit this behavior.

## Results

**Figure 1:**
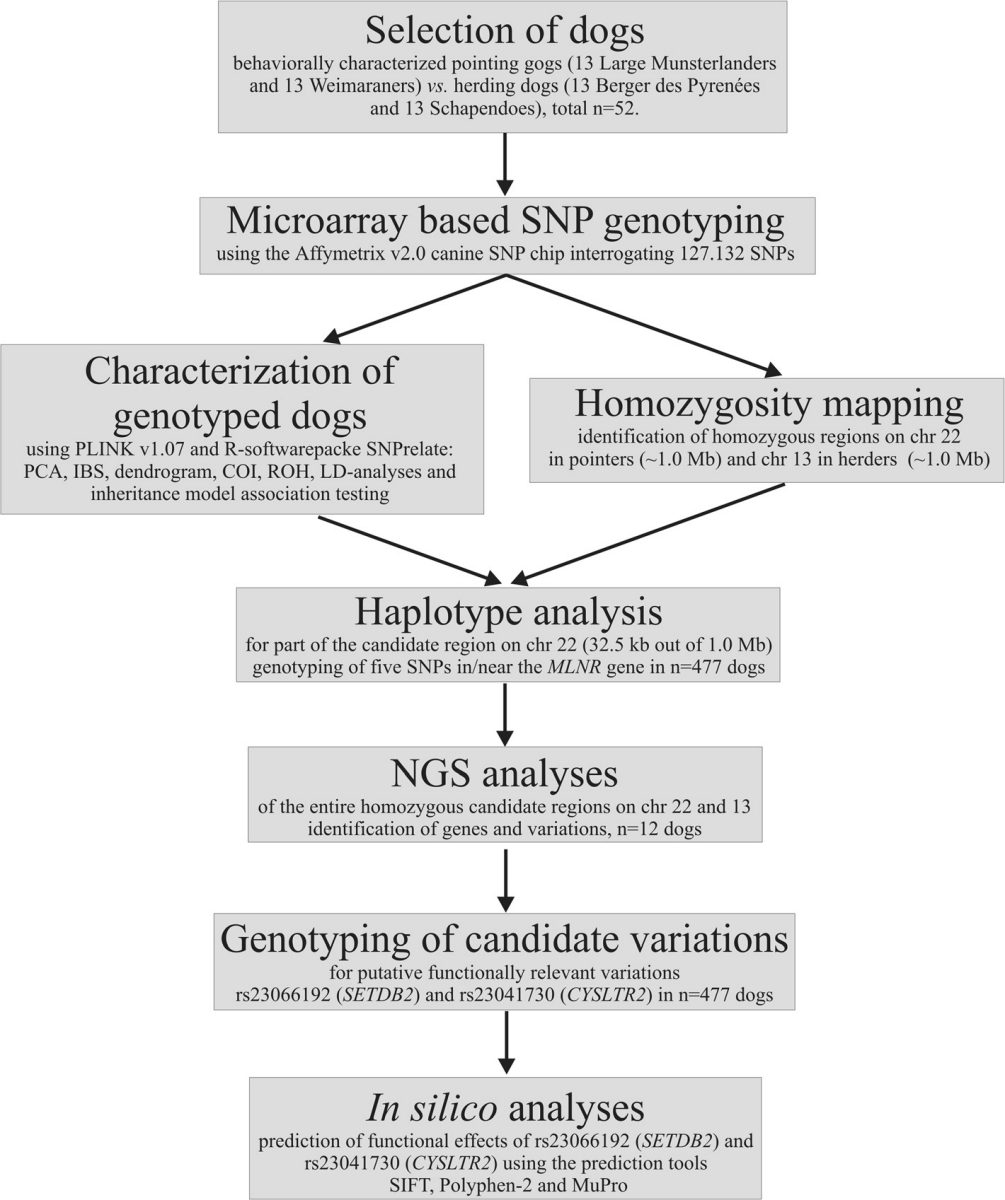
Schematic workflow of the present study.

**Figure 2:**
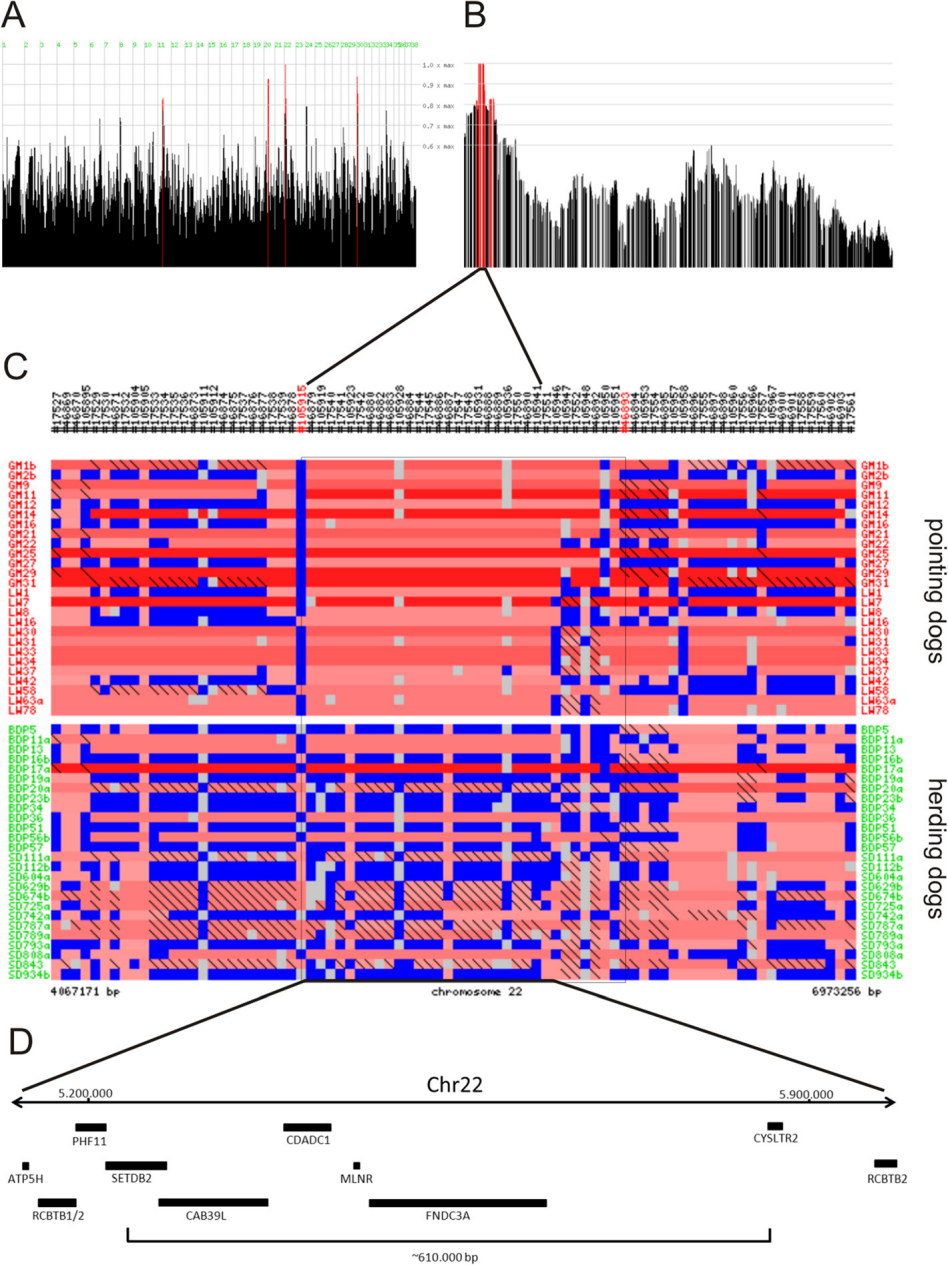
Genome-wide mapping reveals homozygous regions on chromosome 22 in pointing dogs. Genome-wide mapping reveals homozygous regions on chromosome 22 in 26 pointing dogs (GM, Large Munsterlander; LW, Weimaraner) as compared to 26 herding dogs (BDP, Berger des Pyrenées; SD, Schapendoes). Homozygosity peaks were revealed by Homozygosity Mapper analysis in pointing dogs **(A)**. Homozygosity peaks for part of magnified chromosome 22 **(B)**: Genomic areas with homozygosity scores (HS) of >0.8 are depicted in red color. Regions with HS = 1.0 were further analysed in detail. Genotyping results for the corresponding regions are depicted in different colors for the individual dogs (**C**; blue = heterozygous, red = homozygous, dark to light shades of red indicate longer and shorter homozygosity stretches, respectively; gray = unknown; color patterns are predefined by Homozygosity Mapper). Genotypes homozygous for the minor allele are marked with black diagonal bars. Genes included are depicted as black bars including their respective abbreviated designations **(D)**.

**Figure 3:**
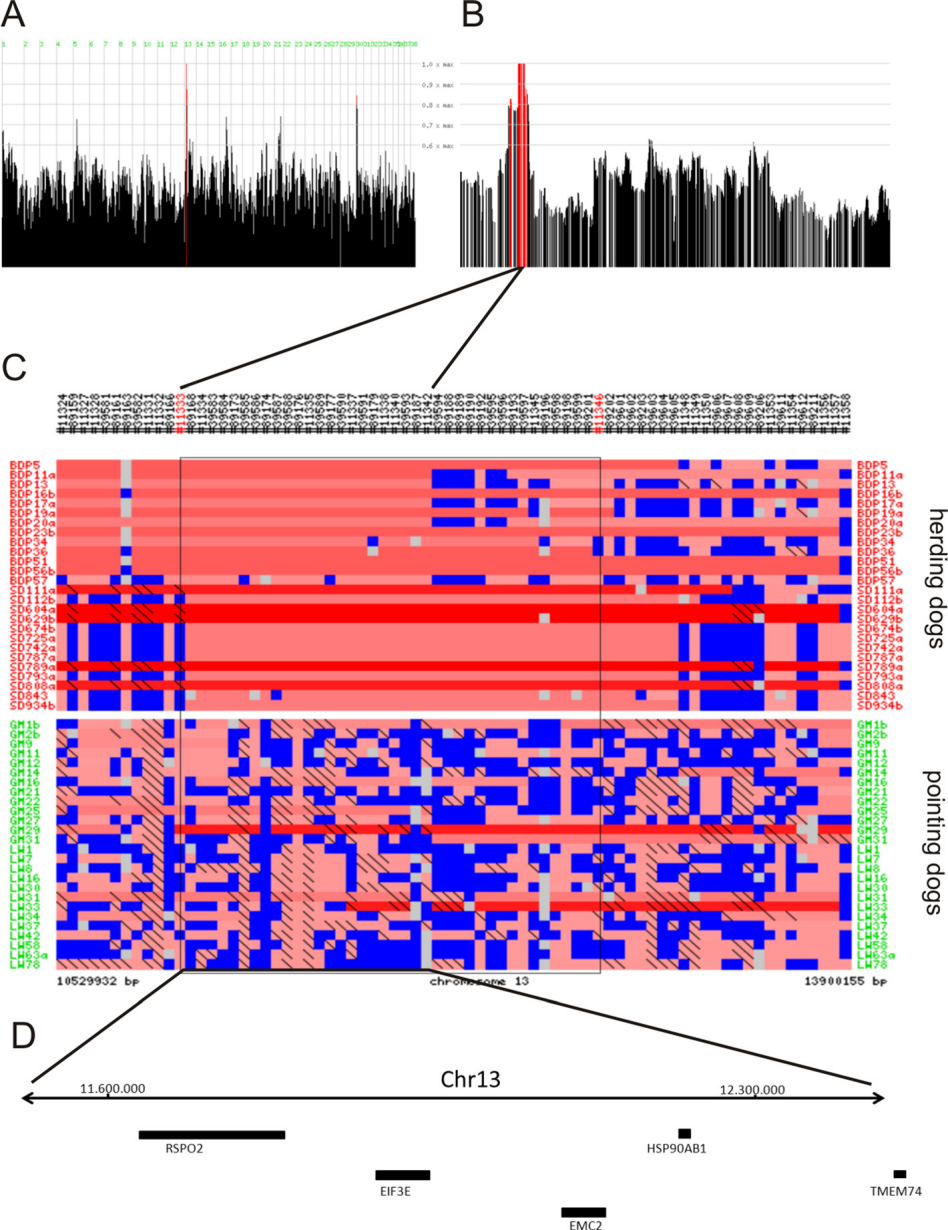
Genome-wide mapping reveals homozygous regions on chromosome 13 in herding dogs. Genome-wide mapping reveals homozygous regions on chromosome 13 in 26 herding dogs (BDP, Berger des Pyrenées; SD, Schapendoes) as compared to 26 pointing dogs (GM, Large Munsterlander; LW, Weimaraner). Homozygosity peaks were revealed by Homozygosity Mapper analysis in pointing dogs **(A)**, homozygosity peaks for part of magnified chromosome 13 **(B)**: Genome areas with homozygosity scores (HS) of >0.8 are depicted in red color. Regions with HS = 1.0 were further analysed in detail. Genotyping results for the corresponding regions are again depicted in colors for the individual dogs (**C**; blue = heterozygous, red = homozygous, dark shades of red indicate longer and light shorter homozygosity stretches, respectively; gray = unknown; color patterns are predefined by Homozygosity Mapper). Genotypes homozygous for the minor allele are marked with black diagonal bars. Genes included in this region are depicted as black bars including their respective abbreviated designations **(D)**.

We undertook a combined homozygosity mapping and genomic sequencing study to genetically compare pointing dogs and herding dogs (see also Table 1), and to identify nucleotide variations specific to pointing dogs; we used herding dogs as controls, as they do not show pointing behavior. In our case–control investigation, using a SNP chip array-based analysis with genotyping and homozygosity mapping (Figure 1; Additional file 1:Table S1), we showed high homozygosity on chromosome 22 in the two pointing breeds Large Munsterlander and Weimaraner compared with the two herding breeds Berger des Pyrenées and Schapendoes (Figure 2). In contrast, a region of extended homozygosity was evident on chromosome 13 in the herding breeds, which could not be detected by homozygosity mapping on the same chromosome in pointing breeds (Figure 3).

**Table 1:** **Dog breeds and corresponding sample numbers (N)**

			**N**					
**Category**	**Breed**	**FCI No.**	**Total**	**SNP-array**	**Haplotype**	**Illumina based NGS**	**Candidate variations**	**Categorized by FCI incl. historical usage**
	German Shorthaired Pointing Dogs	119	20		20		20	pointing/versatile hunting
Pointing dogs (N = 172)	Weimaraners	99	78	13	78	3 out of the 13	78	pointing
	Large Munsterlanders	118	75	13	75	3 out of the 13	75	pointing
	German Longhaired Pointing Dogs	117	7		7		7	pointing/versatile hunting
	Gordon Setters	6	6		6		6	pointing
	Irish Setters	120	5		5		5	pointing
	English Setter	2	1		1		1	pointing
Other hunting dogs (N = 120)	Glen of Imaal Terriers	302	45		45		45	fox and badger hound
	Dachshunds	148	23		23		23	hunting below ground
	Labrador Retrievers	122	21		21		21	retrieving
	Flat Coated-Retrievers	121	2		2		2	retrieving
	Golden Retrievers	111	8		8		8	retrieving
	German Wachtelhunds	104	18		18		18	versatile hunting
	wolves		3		3		3	-
Herding dogs (N = 165)	Bergers des Pyrenées	141	42	13	42	3 out of the 13	42	herding and driving
	Schapendoes	313	68	13	68	3 out of the 13	68	herding and driving
	Giant Schnauzers	181	41		41		41	herding and driving/guarding and companion
	Kuvasz	54	14		14		14	herding and driving
	sum		477	52	477	12	477	

Category corresponds to the behavioral classification; FCI No. = Fédération Cynologique Internationale number of each breed listed.

To characterize individual breeds and establish their genetic relatedness, we assessed the coefficient of inbreeding (COI, Additional file 1: Table S2), runs of homozygosity (ROH, Additional file 1: Table S3), undertook a principal component analysis (PCA, Additional file 1: Figure S1), and measured identity-by-state (IBS, see dendrogram in Additional file 1: Figure S2) and models of inheritance (Additional file 1: Table S4). First, the COI analysis showed comparatively low (4.4% for Berger des Pyrenées and 2.6% for Large Munsterlander) to moderate (14.1% for Schapendoes and 8.5% for Weimaraner) inbreeding coefficient estimates. These findings were in accordance with those of ROH (mean number of homozygous segments per breed values for Berger des Pyrenées: 14.69 ± 6.90, Large Munsterlander: 6.31 ± 2.90, Schapendoes: 24.15 ± 9.25 and Weimaraner: 10.92 ± 4.29); higher COIs were associated with increased numbers of homozygous regions. Results from both the PCA and IBS analyses indicated a limited degree of relatedness among individual dogs of a particular breed, and a clear distinction among the four breeds studied (*i.e.* Berger des Pyrenées, Large Munsterlander, Schapendoes and Weimaraner), as indicated in the cluster dendrogram (Additional file 1: Figure S2). In addition, the IBS analysis showed that Large Munsterlander and Weimaraner are closely related, as are Berger des Pyrenées and Schapendoes, providing strong support for the differentiation of pointing and herding dogs.

**Figure 4:**
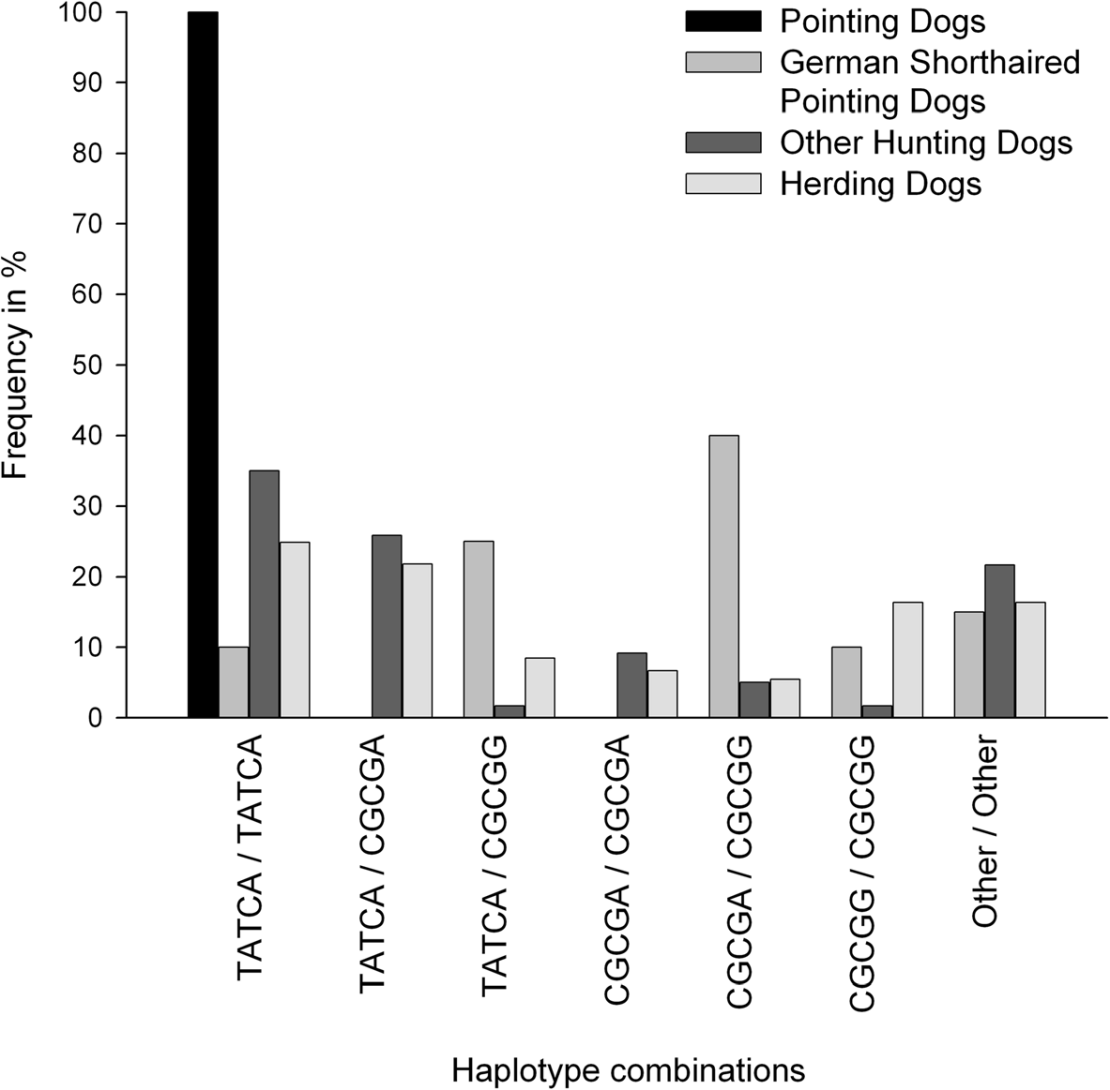
Haplotype frequencies in candidate region on chromosome 22. Frequencies of chromosome 22 haplotype combinations in/near the *MLNR* gene spanning 32.5 kb. Pointing dogs (n = 172; 1 English Setter, 7 German Longhaired Pointing Dogs, 6 Gordon Setter, 5 Irish Setter, 75 Large Munsterlander and 78 Weimaraner dogs), 20 German Shorthaired Pointing Dogs, other hunting dogs including wolves (n = 120; 23 Dachshunds, 2 Flat Coated Retriever, 45 Glen of Imaal Terrier, 8 Golden Retriever, 21 Labrador Retriever, 18 German Wachtelhunds and 3 wolves) and herding dogs (n = 165; 42 Berger des Pyrenées, 41 Giant Schnauzers, 14 Kuvasz and 68 Schapendoes) were compared. Homozygosity for the haplotype TATCA (SNPs rs23039271, rs23029055, rs23064266, rs23039274 and rs23003918) was observed in all pointing dogs except German Shorthaired Pointing Dogs.

Microarray SNP-genotyping of 26 pointing dogs and 26 herding dogs (Table 1; Additional file 1: Table S1) and mapping showed extensive of homozygosity in a ~1.0 megabase (Mb) candidate genomic region on each chromosome 22 (hunting dogs) and chromosome 13 (herding dogs). Homozygosity in additional 192 dogs representing all seven pointing breeds included here (*i.e.* English Setter, German Longhaired Pointing Dog, German Shorthaired Pointing Dog, Gordon Setter, Irish Setter, Large Munsterlander and Weimaraner; see Table 1) was then confirmed in a separate SNP analysis of a small region (32.5 kb) harboring the *MLNR* gene (Figure 4). Specifically, dogs representing six of the seven pointing breeds (*i.e.* excluding German Shorthaired Pointing Dogs) were haplotypic in a homozygous state for this particular region, which was significantly different from herding breeds (Berger des Pyrenées, Giant Schnauzer, Kuvasz and Schapendoes) (p < 0.0001, χ^2^-testing using r × c contingency tables; df = 9) and some hunting breeds (Dachshund, Flat Coated-Retriever, German Wachtelhund, Glen of Imaal Terrier, Golden Retriever, Labrador Retriever and wolf) that do not exhibit pointing behavior (p < 0.0001, χ^2^-testing using r × c contingency tables; df = 9). Table 1 shows the dog breeds used in this study with respect to pointing behavior.

Illumina-based sequencing of the two ~1.0 Mb homozygous regions in the genomes of six pointing dogs (three Large Munsterlander and three Weimaraner) and herding dogs (three Berger des Pyrenées and three Schapendoes) included ten genes (chromosome 22) and five genes (chromosome 13), respectively (Table 2; Additional file 1: Table S1). In the pointing breeds (Large Munsterlander and Weimaraner), we detected 13 homozygous SNPs in coding regions, 5′- or 3′-untranscribed regions (UTRs) of five genes (*CDADC1, CYSLTR2, MLNR, RCBTB1* and *SETDB2*) by comparison with the herding dogs studied (Table 3). Conversely, in selected herding dogs (*i.e.*, Berger des Pyrenées and Schapendoes), we detected four SNPs (in homozygous state) in 5′- or 3′-UTRs of both genes *RSPO2* and *TMEM74* with respect to pointing dogs.

**Table 2:** **Chromosomal regions typed in homozygous state (H.S. score >0.8) in pointing and herding dogs**

	**H.S. score**	**Chromosomal region**	**Size**	**Genes comprised**
Pointing *vs*. herding dogs	1.0	chr22:5,120,014-5,933,118	~1.0 Mb	*ATP5H, RCBTB1, RCBTB2, PHF11,* ***SETDB2*** *, CAB39L, CDADC1, MLNR, FNDC3A,* ***CYSLTR2***
Pointing *vs*. herding dogs	1.0	chr13:11,505,788-12,451,615	~1,0 Mb	***RSPO2*** *, EIF3E, TTC35, EMC2, HSP90AB1,* ***TMEM74***

Candidate regions chosen for subsequent NGS analyses were defined as regions showing recurrent hits surpassing the 0.8 threshold using the program Homozygosity Mapper for the three analyzed file sets (see also Table S5). Primary candidate regions were narrowed down by selection of regions uniformly comprising 100% homozygosity. Candidate genes containing potential behaviorally relevant sequence variations as identified by NGS analysis are highlighted in bold.

**Table 3:** **Variations in coding and flanking regions in candidate genes with corresponding physical positions according to the reference sequence**

	**Gene**	**Chromosome**	**Position**	**Reference sequence**	**Variation**	**Amino acid exchanges/ non-coding regions**	**rs number (build 131)**
Pointing *vs*. herding dogs	*RCBTB1*	22				3′UTR	
	*RCBTB1*	22	5154333	T	C	F > F	rs23035959
	*PHF11*	22				3′UTR	
	*SETDB2*	22				5′UTR	
	***SETDB2***	**22**	**5213748**	**C**	**T**	**S** ^#^ **> N**	**rs23066192**
	*SETDB2*	22				3′UTR	
	*CDADC1*	22	5391565	C	T	V > V	-
	*CDADC1*	22				3′UTR	
	*MLNR*	22	5451915	G	C	V > V	rs23039274
	*FNDC3A*	22				3′UTR	
	*CYSLTR2*	22				5′UTR	
	*CYSLTR2*	22	5859501	A	G	L > L	rs23041728
	***CYSLTR2***	**22**	**5860286**	**C**	**G**	**P > R** ^**#**^	**rs23041730**
	*RSPO2*	13				5′UTR	
Herding *vs*. pointing dogs	*RSPO2*	13				3′UTR	
	*TMEM74*	13				5′UTR	
	*TMEM74*	13				3′UTR	

Corresponding rs identifiers for the sequence variations are listed if available. Sequence variations resulting in amino acid substitutions are indicated in bold. # = The amino acid present in pointing dogs. (Reference sequences from UCSC genome browser, May 2005; Broad/canFam2)

Of all 17 SNPs detected, two (*i.e.* rs23041730 and rs23066192) were non-synonymous (Table 3). In pointing dogs, these two SNPs were specifically linked to individual amino acid exchanges in the proteins encoded by genes *CYSLTR2* and *SETDB2*, respectively. Bioinformatic analyses (using the program PolyPhen-2 [14]) indicated that the non-synonymous SNP rs23041730 (Pro > Arg) in the *CYSLTR2* gene might affect the functionality of the gene product (score 0.938), although SNP rs23066192 (Ser > Asn) in the *SETDB2* gene (transcripts ENSCAFT00000044150 and ENSCAFT00000006968) was predicted not to affect the functionality of its encoded protein. SIFT [15] entries obtained from the Ensembl [16] database for the two non-synonymous exchanges indicate borderline tolerance of the rs23041730 exchange, with a score of 0.06 for the *CYSLTR2* gene and tolerance of the rs23066192 exchange, with scores of 0.23 and 0.32 for the *SETDB2* gene.

However, the stability of the protein encoded by the *SETDB2* gene was predicted (by MUpro) to increase through a Ser > Asn change (rs23066192, confidence score 0.46), whereas it decreased for a Pro > Arg change (*CYSLTR2,* rs23041730, confidence score: −0.49). As these analyses suggested at least in part a functional consequence of SNP rs23066192 in *SETDB2* and SNP rs23041730 in *CYSLTR2*, we then showed consistent homozygosity for both SNPs in additional pointing dogs (n = 166, six breeds) representing six of the seven pointing breeds (*i.e.* excluding German Shorthaired Pointing Dogs) and established linkage equilibrium in herding dogs (n = 165, four breeds) as well as in other hunting dogs without pointing behavior (n = 120, 6 breeds and wolves) (Additional file 1: Table S6). An alignment of the amino acids sequences inferred from genes *CYSLTR2* and *SETDB2* to their respective orthologs in other mammalian species revealed that the Pro > Arg alteration (SNP rs23041730) is located in the extracellular domain of CYSLTR2 protein and the Ser > Asn change (SNP rs23066192) is within the SET domain of SETDB2, respectively.

## Discussion

In the present study, we utilized a total of 66,164 markers for subsequent mapping, ensuring sufficient genome coverage. Usually fewer markers are employed for genome-wide association studies (GWAS) in dogs than in humans, which relates to an extended linkage disequilibrium in regions of dog genomes of megabases compared with kilobases for human genomes [11,17]. In our study, we calculated similar degrees of r^2^ decay over distance among breeds including hunting and herding dogs (Additional file 1: Figure S3). Thus, although many genomic markers were excluded, our data provided a solid basis of further analyses. In comparison with previous studies, we used a comparable number of markers ranging from 43 times more [2] or 1/3 [18], respectively. Therefore, it is possible that some candidate genes might not have been detected, since many regions comprised fewer markers (Additional file 1: Figure S4 and Figure S5). Nevertheless, the study of Vaysse *et al.* [18] identified similar genomic regions on chromosomes 13 and 22 in different dog breeds including herding and hunting dogs.

Our comparison of two herding vs. two pointing breeds identified a region of extended homozygosity on chromosome 13 harboring the candidate genes *RSPO2* and *TMEM74* for the herding dogs. However, neither coding variations nor exchanges in flanking regions were identified by NGS for these genes. Given the similar coat types of the herding dogs in our study, it is likely that we replicated the association of the *RSPO2* with hair-growth phenotypes in dog breeds [18,19]. In addition, *RSPO2* was reported to play a role in the refinement of patterning in the mammalian cochlea [20]. *TMEM74* plays a crucial role in autophagy induced by starvation [21] and thus perhaps also in the manifestation of cancer, neurodegenerative diseases and cardiomyopathies [22]. A direct link to herding behavior is therefore not obvious.

In our work the results of Vaysse *et al.* [18] could be confirmed and further analyzed in detail for chromosome 22 with regard to hunting/pointing breeds. Comparing two hunting vs. two herding breeds we identified a region on chromosome 22 harboring amongst others the candidate genes *CYSLTR2* and *SETDB2* (Table 2). Compared to our study, Vaysse *et al.* [18] identified a smaller region on chromosome 22 that is shared amongst eight breeds and contains the *FNDC3A* and *CYSLTR2* gene. When comparing the two studies the basic difference lies in between the breeds and comparison criteria used. Vaysse *et al.* [18] applied across-breed GWAS and examined overlapping genomic regions exhibiting high levels of differentiation between dog breeds, independent of breed categories, with respect to morphological and behavioral traits defined as by boldness, drop ear size, weight, furnish, tail curl, curiosity/fear, aggression, sociability, chase-proneness and playfulness. Interestingly, the identified region on chromosome 22 by Vaysse *et al.* [18] was shared by eight dog breeds most of which have an history of hunting (Beagle: foxhound; Border Terrier: fox and vermin hound; English Bulldog: bull baiting; Gordon Setter: setter/pointing dog; Irish Wolfhound: wolf and bear hound; Newfoundland: working dog; Rottweiler: herding livestock; Weimaraner: pointing dog).

When looking at those dogs which have a proven history record of pointing only two of the eight dog breeds remain, namely the Gordon Setter and the Weimaraner. For both breeds, Vaysse *et al.* [18] identified the genomic region on chromosome 22 harboring the *CYSLTR2* gene and, in line with our results, the *SETDB2* gene. Therefore, it may be speculated that the overall comparison by Vaysse *et al.* [18] identified a concise region important for the hunting trait, and that the herein identified region harboring both genes, *CYSLTR2* and *SETDB2*, may be a prerequisite for the trait for pointing behavior. Our approach involved dogs with individually verified pointing behavior, in contrast to Vaysse *et al.* [18], where multiple breeds were compared. Although both studies share similar results, the study designs addressed different purposes, but partly complement each other.

The SNPs rs23041730 and rs23066192 represent variations in the coding genes *CYSLTR2* and *SETDB2*, respectively, and were consistently fixed for six of seven pointing dog breeds (excepting German Shorthaired Pointing Dogs) compared with dogs that do not point, and are thus proposed to contribute, at least in part, to canine pointing behavior. The functional consequence of the mutation in the extracellular domain of CYSLTR2 is not presently known, but it might influence ligand binding of the leukotrienes LTC_4_, LTD_4_ and LTE_4_ [23]. Although CYSLTR2 is interpreted to be less likely to relate to behavioral phenotype, because of its immunological role in humans [24], its possible involvement needs to be evaluated experimentally. By contrast, proteins that contain a SET domain are known to modulate gene expression epigenetically via histone H3 methylation in humans [25]. Thus, SETDB2 could be a histone H3 methyltransferase, as it contains an active site and key flanking cysteine residues required for catalytic activity [26]. Interestingly, SETDB2 is also associated with the establishment of left-right asymmetry [27]. Since the degree of motor laterality has been identified as a key predictor of success in guide-dog training [28], it can be speculated that a variation in *SETDB2* might be associated with learning abilities that in turn might influence the establishment of pointing behavior.

Given that behavioral traits, such as pointing, likely depend on multiple genetic components [29], mutations in *CYSLTR2* and/or *SETDB2* might play a role or at least contribute to a breed-specific behavior. However, it is possible that other genes might be involved in expressing the pointing trait, since complex behavioral patterns are not always explicable by the effect(s) of a single gene. It might be that multiple genes contributing to a particular behavior are spread across the chromosomal complement and are located in regions with lower homozygosity scores (<1.0; see Additional file 1: Table S6). However, it is also possible that variations in non-coding regions might play a role e.g. resulting in cryptic splice sites or altered miRNA binding domains. Therefore, although we predict here that a candidate region on chromosome 22 associates with pointing behavior, other genomic regions (coding and/or non-coding) could also contribute to this phenotype. Future multi-breed studies, encompassing additional pointing breeds in combination with higher density SNP arrays, may further contribute to clarify the genetic basis of the pointing trait.

The complexity of the relationship between behavior and genotype is exemplified by the discrepancy in results between studies. While a previous study of 147 dog breeds [2] suggested candidate genomic regions for pointing and herding behavior, our data did not show any concordance with findings from this study, likely due to the different methodologies employed. First, Chase *et al*. [2] used 1,536 SNPs, while we employed > 66,000 markers in our study. Second, Chase *et al*. [2] assumed the behavioral phenotype following consultation with an experienced dog trainer who categorized the breeds in general, whereas in the present study, we verified pointing behavior in each individual dog based on official examinations. Clearly, future genomic analyses should focus sharply on making the connection between the phenotype and genotype for pointing through selective breeding of different pointing and non-pointing breeds and typing for variations in *SETDB2* and *CYSLTR*.

Another interesting study might involve transcriptomic and proteomic investigations of the dog brain, focusing on specific brain areas establishing the functional relevance of SNPs and associated amino acid changes [30]. Interestingly, although pointing and herding represent distinct behavioral phenotypes, some traits such as smelling and attention to animal targets are shared among different dog breeds. Therefore, there is considerable scope for fundamental investigations of the underlying biological and behavioral processes linked to a particular genetic background.

More generally, archetypal pointing behavior can be regarded as a response to an external stimulus that can be exhibited in hunting dogs without specific training for hunting, rendering it an ideal trait for genetic studies. The German Shorthaired Pointer represents an exception in our study because both amino acid exchanges in the *SETDB2* and *CYSLTR2* genes were not consistently present as in the other pointing breeds. Nevertheless, this fact does not exclude these candidate genes contributing to pointing, because Parker et al. [31] studied genetic differentiation of dog breeds with microsatellite markers. Most of the 85 breeds studied form distinct clusters except of four, the latter including the German Shorthaired Pointer. This breed has been heavily influenced by English Pointers in North America, but also in Europe. Thus the within group variation is higher than expected and might be the cause of this exception. Disregarding the deviation of the German Shorthaired Pointing Dogs, two groups of hunting dogs can be classified: those with and those without pointing behavior. The hunting dogs that do not point include the German Wachtelhund, which represents a flushing breed, also called the *Stoeberer* (“rummager”; FCI-Standard No 104). Interestingly, the genotyping results for *SETDB2* and *CYSLTR2* revealed heterozygous status for this breed. The Wachtelhund is also derived from the same *Hühnerhund* or spaniel-type stock as the Large Munsterlander and Weimaraner but it remained a flushing and not a pointing dog. In this breed, pointing behavior can be trained, at least to some extent (Dr Helga Adolph, personal communication). It might be speculated that heterozygous genotypes in this breed might contribute to a lesser extent to pointing behavior than homozygous ones, and might serve as a basis for to assess how pointing can be influenced by other factors, such as environment and training.

## Conclusions

We submit that the candidate region on chromosome 22 is, at least partly, relevant for the trait of pointing. Detailed breeding experiments may yield deeper insights into the genetics of this trait, yet large-scale crossings herald potential ethical conflicts. Thus, combined genetic and (quantitative) behavioral analyses appear warranted, for instance, with respect to the regulatory elements comprised in this genomic region.

## Methods

### Samples

Genomic DNAs were isolated from peripheral blood leukocyte or buccal swab samples from 477 individual dogs using a standard protocol [32]. The quality of each DNA sample was verified by agarose gel electrophoresis. All canine DNA samples comprised in this study were either submitted to DNA biobanks (for all of all hunting dogs established in our institute) or were sent to our institution based on the request for analysis of monogenetic diseases (e.g. progressive retinal atrophy). In both cases informed consent was obtained from all dog owners for sample collections and all genetic investigations. In each case sample collection was performed by board-certified veterinarians according to international guidelines for the use of laboratory animals. As the DNA stems from the blood of client-owned dogs that underwent routine veterinary examinations including venipuncture, no “animal experiments” were performed, and approval by an ethical committee was not necessary.

All pointers passed official examinations (*i.e. Verbandsjugendprüfung*, *Herbstzuchtprüfung* and/or *Verbandsgebrauchsprüfung*; http://www.jghv.de/Prüfungsordnungen) for their pointing behavior. In Germany and some other German-speaking countries, such examinations must be passed for any hunting dog to be registered for breeding. A summary of the breeds studied here, and corresponding sample numbers are given in Table 1. In brief, 52 dogs (13 of each Berger des Pyrenées, Large Munsterlander, Schapendoes and Weimaraner) were used for homozygosity screening. The genomes of three dogs of each of these four breeds were sequenced using Illumina technology and sequence variations explored for the entire dog cohort.

### Microarray-based genotyping

Dogs were selected based on their pointing (Large Munsterlander and Weimaraner) or herding (Berger des Pyrenées and Schapendoes) behavior. For each of these four breeds, 13 dogs were selected for genotyping using the Affymetrix v2.0 canine single nucleotide polymorphism (SNP) chip array (max. 127,132 SNPs). Dogs with as few as possible common predecessors were selected (from different kennels) based on detailed pedigree analyses, to ensure minimum genetic relatedness. In order to avoid possible gender-related factors, 27 male and 25 female dogs (see Additional file 1: Table S1) were selected for microarray-based genotyping, conducted following the manufacturer’s protocol (Affymetrix). Genotyping calls were made using Affymetrix Power Tools employing the BRLMM-P algorithm and are available under the Additional file 2. All arrays surpassed the recommend genotype call rates of greater than 75% (Additional file 1: Table S1; Additional file 3). Due to the known limitation of over-calling heterozygous genotypes using the Array v.2 full set (127,132 SNPs) [33], subsequent homozygosity screening, cluster dendrogram, linkage disequilibrium (LD) as well as principle component analysis (PCA) were performed on the filtered data sets. In brief, we used a total of three different file sets, in order to reliably identify homozygous genomic regions. The first analysis file consisted of the Array v.2 platinum set, a validated subset of the entire chip, which contained 49,663 SNPs high-quality SNPs (http://www.broadinstitute.org/mammals/dog/caninearrayfaq.html). The second file represented a data set filtered from the initial Array v.2 full set (127,132 SNPs) using the program PLINK v.1.07 [34]. Filtering criteria included a maximum SNP ‘missingness’ rate (as defined by PLINK) of 40% per locus, a maximum individual missingness rate of 100% (indicating that all individuals were analyzed) and a Hardy-Weinberg threshold of 0.05, resulting in a file set of 66,915 SNPs. The third file consisted of the PLINK-filtered data which were also filtered to exclude SNPs with a heterozygosity rate of >60% per locus (the threshold was applied to exclude genotyping errors for the expected maximum heterozygosity rate of 50%) for all analyzed dogs, leading to a set of 66,164 SNPs.

### Statistical analyses

Statistical analyses were conducted on the PLINK-filtered data set where marker showing excess of heterozygosity (>60%) over all breeds were excluded (66,164 SNPs). PCA and identity-by-state (IBS) measurements, with corresponding dendrogram clustering, were performed using SNPRelate [35], a statistical package in R. Coefficient of inbreeding (COI), runs of homozygosity (ROH), inheritance model association testing, linkage disequilibrium (LD) analysis, and the calculation of the physical distribution and distance between two neighboring markers were assessed using PLINK employing default settings. Supplementary data are available in Additional file 1: Tables S2-S5 and Figures S1-S5.

### Homozygosity mapping

Our investigative approach is based on the premise that alleles responsible for a particular trait should exist in homozygous state due to selective (in)breeding over many generations [36]. Here, homozygosity was investigated using the publicly available program Homozygosity Mapper (see http://www.homozygositymapper.org for detailed information) [37]. We used a total of three different file sets to reliably identify homozygous regions. The comparison of pointing and herding dogs, and *vice versa*, was performed using a conventional model and a block-length limit of 80 for a marker set of n > 45,000, accordingly to our filtered marker set of 66,164 SNPs. The threshold for the homozygosity score was set at > 0.8 (which shows all scores of > 80% of the maximum score detected in the analysis). Inspection of the genomic regions exceeding the 0.8 threshold for each data file analyzed and comparison (hunting *vs*. herding, and *vice versa*) revealed that some of the calls contained only breed-specific and not “category”-specific (pointing *vs*. herding) homozygous regions (Additional file 1: Table S6). Primary candidate regions were selected based on concordant hits for all three datasets for which both breeds of one category exhibited homozygosity in comparison to the other. The results are depicted in Table 2. Based on dog genome data (UCSC genome browser, May 2005; Broad/canFam2) available at the time of analysis, the *MLNR* gene was the sole gene mapped to the homozygous candidate region identified on chromosome 22 in the reference sequence tract (UCSC genome browser, May 2005; Broad/canFam2). As proof-of-principle for the genotyping results from genotyping arrays and homozygosity mapping analysis, two originally genotyped SNPs with the Affymetrix v.2.0 canine SNP chip (rs23003918 on chr22:5483300 and rs23039271 on chr22:5444775) and three adjacent additional SNPs (rs23039274 on chr22:5451915; rs23064266 on chr22: 5451557; and rs23029055 on chr22:5450817) were selected for genotyping by restriction fragment length polymorphism (RFLP) analysis. All five markers were analyzed in the initially-typed 52 dogs and additional 425 dogs from several breeds (see Table 1).

### Next generation sequencing by Illumina technology

Candidate regions identified *via* homozygosity mapping were selected for sequencing (Table 2). Sequence analyses and detection of variations (including SNPs) were conducted by ATLAS (Biolabs, GmbH). Briefly, target-enrichment for the entire candidate regions was achieved using Illumina TruSeq DNA Library Preparation (NimbleGen SeqCap EZ library, including intronic and exonic regions). Sequencing was performed on an Illumina HiSeq 2000 using a single lane per sample, generating paired-end reads of 2x100 nt in length and yielding a mean of 460 ± 70 x coverage. Subsequent alignment and indexing were performed using BWA1 and SamTools2 (http://samtools.sourceforge.net/), respectively [38]. The program GATK3 (http://www.broadinstitute.org/gatk/) [39] was used for the detection of InDels and SNPs. The dog (*Canis familiaris*) whole genome shotgun (WGS) assembly v.2.0 May 2005 (available at http://genome.ucsc.edu/) served as the reference.

For each breed used for initial homozygosity mapping, three dogs of each of the four breeds (total n = 12) were selected for sequencing. SNPs were investigated using the Integrative Genomics Viewer (IGV; http://www.broadinstitute.org/igv/) [40,41]. The xenoRefGene.txt track from UCSC (http://hgdownload.soe.ucsc.edu/goldenPath/canFam2/database/) was used to identify genes in the candidate genomic regions through an alignment of the canine DNA sequence to RefSeqs from other mammalian species, in order to validate whether indels and/or SNPs were located in coding or neighboring flanking regions (±5 bp) of genes.

Candidate nucleotide variations were those that related to consistent homozygous calls for all dogs in the corresponding case group compared with heterogeneous genotype calls in the control group. Primary candidate regions were defined as coding regions, including flanking introns. Further candidate regions were defined as 5′- and 3′-untranslated regions (UTR). Such variations and their corresponding flanking sequences were compared by BLASTn analysis against the canine genome to assess whether pseudogenes or homologous regions had been falsely enriched during library preparation. Variations in pseudogenes or non-coding regions were excluded. The most promising candidate variations were genotyped via RFLP in the initially sequenced 12 dogs and additional 465 dogs from several breeds (see Table 1).

### *In silico* analyses

Damaging effects of sequence variations were interpreted using the web-based prediction program PolyPhen-2 [14] and the SIFT entries of the ensembl database (http://www.ensembl.org/) [16]. Protein stability was predicted with the MUpro algorithm [42]. Sequences were aligned and compared using the UniProt database (The UniProt Consortium, available at http://www.uniprot.org/). In order to predict the functional roles of candidate genes, we scrutinized multiple databases: National Center for Biotechnology Information (NCBI; http://www.ncbi.nlm.nih.gov/), GeneCards (http://www.genecards.org/), the Neuroscience Information Framework - NIF (https://www.neuinfo.org/), the Mouse Genome Informatics- MGI (http://www.informatics.jax.org/), University of California Santa Cruz – UCSC Genome Browser (https://genome.ucsc.edu/), ENSEMBL (http://www.ensembl.org/index.html) and UniProt (http://www.uniprot.org/).

### Availability of supporting data

The data sets supporting the results of this article are included within the article and additional files or are available upon request.

## Additional files

Additional file 1:
**Contains following supporting tables and figures.** **Table S1.** Dogs investigated using the GeneChip Canine Genome 2.0 Array and Next Generation Sequencing (NGS). **Table S2.** Inbreeding coefficient analysis for the genotyped dogs. **Table S3.** Runs of homozygosity using PLINK analysis. **Table S4.** Alternate/full model association tests using PLINK for the two candidate SNPs rs23066192 (*SETDB2* gene) and rs23041730 (*CYSLTR2* gene). **Table S5.** Haplotype frequencies for SNPs combinations rs23041730 and rs23041728 in the *CYSLTR2* gene and rs23066192 and rs23041730 in the *SETDB2* and *CYSLTR2* genes. **Table S6.** Homozygous genomic regions identified by Homozygosity Mapper. **Figure S1.** Principle Component Analysis (PCA). **Figure S2.** Cluster dendrogram. **Figure S3.** Average r^2^ decay plot. **Figure S4.** Chromosomal SNP distribution. **Figure S5.** Median marker distance. Additional file 2:
**Microarray genotype brlmm-p.calls based on the Affymetrix Power Tool for the 52 analyzed dogs.**
Additional file 3:
**Microarray genotype brlmm-p.report call rate for the 52 analyzed dogs.**

## Abbreviations

COI: Coefficient of inbreeding
DNA: Deoxyribonucleic acid
GWAS: Genome wide association study
IBS: Identity-by-state
Mb: Megabase
NGS: Next generation sequencing
PCA: Principal component analysis
RFLP: Restriction fragment length polymorphism
ROH: Runs of homozygosity
SIFT: Sorts intolerant from tolerant
SNP: Single nucleotide polymorphism
UTR: Untranslated region
