# Supplementary material for: Homozygosity mapping and sequencing identify two genes that might contribute to pointing behavior in hunting dogs
Source: Canine Genet Epidemiol. 2015 Apr 18;2:5. doi: 10.1186/s40575-015-0018-5 (PMC4579392; doi:10.1186/s40575-015-0018-5)
Supplement: Additional file 1: — Contains following supporting tables and figures. Table S1. Dogs investigated using the GeneChip Canine Genome 2.0 Array and Next Generation Sequencing (NGS). Table S2. Inbreeding coefficient analysis for the genotyped dogs. Table S3. Runs of homozygosity using PLINK analysis. Table S4. Alternate/full model association tests using PLINK for the two candidate SNPs rs23066192 (SETDB2 gene) and rs23041730 (CYSLTR2 gene). Table S5. Haplotype frequencies for SNPs combinations rs23041730 and rs23041728 in the CYSLTR2 gene and rs23066192 and rs23041730 in the SETDB2 and CYSLTR2 genes. Table S6. Homozygous genomic regions identified by Homozygosity Mapper. Figure S1. Principle Component Analysis (PCA). Figure S2. Cluster dendrogram. Figure S3. Average r2 decay plot. Figure S4. Chromosomal SNP distribution. Figure S5. Median marker distance. [file 40575_2015_18_MOESM1_ESM.docx]

| breed | **IID** | **gender** | **males/females**  **per breed** | **genotyping** | | | | | | **NGS** |
| --- | --- | --- | --- | --- | --- | --- | --- | --- | --- | --- |
|  |  |  |  | **call rate** | **mean call rate/breed ± SD** | **het rate** | **mean het rate/breed ± SD** | **hom rate** | **mean hom rate/breed± SD** |  |
| Weimaraner | LW78 | female | 4/9 | 84.8 | 85.0±0.3 | 46.4 | 45.0±1.0 | 38.4 | 39.9±1.1 |  |
|  | LW63a | male |  | 84.7 |  | 46.0 |  | 38.7 |  |  |
|  | LW58 | female |  | 84.6 |  | 46.9 |  | 37.6 |  |  |
|  | LW42 | female |  | 85.3 |  | 45.7 |  | 39.7 |  |  |
|  | LW37 | female |  | 85.2 |  | 45.8 |  | 39.4 |  |  |
|  | LW34 | female |  | 84.9 |  | 44.1 |  | 40.7 |  | X |
|  | LW33 | male |  | 84.8 |  | 44.1 |  | 40.8 |  |  |
|  | LW31 | female |  | 85.4 |  | 45.7 |  | 39.7 |  |  |
|  | LW30 | female |  | 85.4 |  | 44.1 |  | 41.3 |  | X |
|  | LW16 | female |  | 84.8 |  | 44.8 |  | 39.9 |  | X |
|  | LW8 | female |  | 85.5 |  | 44.7 |  | 40.8 |  |  |
|  | LW7 | male |  | 84.6 |  | 44.3 |  | 40.3 |  |  |
|  | LW1 | male |  | 85.2 |  | 44.1 |  | 41.1 |  |  |
| Large Munsterlander | GM31 | female | 6/7 | 85.1 | 85.3±0.4 | 45.2 | 46.5±0.8 | 39.9 | 38.8±1.0 |  |
|  | GM29 | female |  | 85.2 |  | 46.6 |  | 38.6 |  |  |
|  | GM27 | female |  | 85.4 |  | 46.6 |  | 38.9 |  |  |
|  | GM25 | male |  | 85.1 |  | 45.9 |  | 39.2 |  |  |
|  | GM22 | female |  | 84.9 |  | 47.8 |  | 37.1 |  |  |
|  | GM21 | male |  | 84.8 |  | 46.4 |  | 38.4 |  | X |
|  | GM16 | female |  | 84.7 |  | 47.7 |  | 37.0 |  |  |
|  | GM14 | male |  | 84.8 |  | 46.4 |  | 38.4 |  | X |
|  | GM12 | female |  | 85.9 |  | 46.7 |  | 39.3 |  |  |
|  | GM11 | male |  | 85.5 |  | 46.1 |  | 39.4 |  |  |
|  | GM9 | female |  | 85.9 |  | 45.1 |  | 40.8 |  | X |
|  | GM2b | male |  | 85.5 |  | 46.7 |  | 38.8 |  |  |
|  | GM1b | male |  | 85.4 |  | 46.8 |  | 38.6 |  |  |
| Schapendoes | SD843 | male | 10/3 | 83.8 | 84.8±+0.4 | 42.3 | 43.1±1.0 | 41.5 | 41.7±1.1 |  |
|  | SD934b | male |  | 85.2 |  | 43.5 |  | 41.8 |  |  |
|  | SD808a | male |  | 85.1 |  | 43.5 |  | 41.6 |  |  |
|  | SD793a | male |  | 85.0 |  | 43.3 |  | 41.8 |  |  |
|  | SD789a | male |  | 84.7 |  | 44.8 |  | 40.0 |  |  |
|  | SD787a | male |  | 85.1 |  | 43.1 |  | 42.0 |  |  |
|  | SD742a | male |  | 85.5 |  | 41.3 |  | 44.2 |  | X |
|  | SD725a | female |  | 85.0 |  | 44.1 |  | 40.9 |  | X |
|  | SD674b | male |  | 85.0 |  | 43.7 |  | 41.4 |  |  |
|  | SD629b | male |  | 84.9 |  | 43.0 |  | 42.0 |  | X |
|  | SD604a | female |  | 84.5 |  | 41.2 |  | 43.3 |  |  |
|  | SD112b | female |  | 84.5 |  | 43.9 |  | 40.5 |  |  |
|  | SD111a | male |  | 84.6 |  | 43.3 |  | 41.3 |  |  |
| Berger des Pyrenées | BDP57 | female | 7/6 | 85.0 | 85.3±0.2 | 46.1 | 45.4±1.0 | 39.0 | 39.8±1.1 |  |
|  | BDP56b | male |  | 85.2 |  | 45.1 |  | 40.0 |  |  |
|  | BDP51 | male |  | 85.4 |  | 45.0 |  | 40.5 |  |  |
|  | BDP36 | female |  | 84.9 |  | 46.6 |  | 38.3 |  |  |
|  | BDP34 | female |  | 85.0 |  | 45.8 |  | 39.1 |  | X |
|  | BDP23b | male |  | 85.2 |  | 46.4 |  | 38.8 |  | X |
|  | BDP20a | female |  | 85.1 |  | 44.7 |  | 40.4 |  |  |
|  | BDP19a | male |  | 85.2 |  | 46.0 |  | 39.2 |  |  |
|  | BDP17a | male |  | 85.4 |  | 43.2 |  | 42.1 |  |  |
|  | BDP16b | male |  | 85.4 |  | 44.0 |  | 41.3 |  | X |
|  | BDP13 | female |  | 85.5 |  | 46.2 |  | 39.3 |  |  |
|  | BDP11a | female |  | 85.7 |  | 45.7 |  | 39.9 |  |  |
|  | BDP5 | male |  | 85.6 |  | 45.6 |  | 40.0 |  |  |

**Table S1. Dogs investigated using the GeneChip Canine Genome 2.0 Array and Next Generation Sequencing (NGS).** From left to right: individual ID (IDD), gender, gender distribution per breed, individual genotyping call rates for the “Array version 2 full set” (127,132 SNPs) utilizing the BRLMM-P algorithm (call rate), mean genotyping call rate per breed ± standard deviation (SD) (mean call rate/breed), individual heterozygosity call rates for the “Array version 2 full set” (127,132 SNPs) utilizing the BRLMM-P algorithm (het rate), mean heterozygosity call rate per breed ± SD (mean het rate/breed± SD), individual homozygosity call rates for the “Array version 2 full set” (127,132 SNPs) utilizing the BRLMM-P algorithm (hom rate), mean homozygosity call rate per breed ± SD (mean hom rate/breed ± SD) and the dogs processed for NGS analysis selected based on passing DNA quality criteria.

| **breed** | **IID** | **E(hom)** | **E(hom)** | **N(NM)** | **F** | **mean F/breed ± SD** |
| --- | --- | --- | --- | --- | --- | --- |
| Weimaraner | LW78 | 40882 | 39720 | 60112 | 0.05699 | 0.085±0.033 |
|  | LW63a | 40968 | 39760 | 60164 | 0.05921 |  |
|  | LW58 | 39746 | 39620 | 59961 | 0.00637 |  |
|  | LW42 | 42057 | 40390 | 61132 | 0.08016 |  |
|  | LW37 | 41610 | 39760 | 60178 | 0.09068 |  |
|  | LW34 | 42623 | 40350 | 61082 | 0.10970 |  |
|  | LW33 | 42235 | 40600 | 61448 | 0.07843 |  |
|  | LW31 | 41296 | 39910 | 60412 | 0.06776 |  |
|  | LW30 | 43496 | 40770 | 61726 | 0.12990 |  |
|  | LW16 | 42227 | 40090 | 60664 | 0.10370 |  |
|  | LW8 | 42470 | 40070 | 60668 | 0.11640 |  |
|  | LW7 | 41471 | 39670 | 60034 | 0.08843 |  |
|  | LW1 | 43212 | 40800 | 61757 | 0.11490 |  |
| Large Munsterlander | GM31 | 42374 | 40300 | 60989 | 0.10020 | 0.026±0.031 |
|  | GM29 | 40976 | 40250 | 60908 | 0.03513 |  |
|  | GM27 | 41247 | 40550 | 61377 | 0.03339 |  |
|  | GM25 | 41056 | 40620 | 61472 | 0.02086 |  |
|  | GM22 | 39631 | 40050 | 60606 | -0.02049 |  |
|  | GM21 | 40775 | 39840 | 60276 | 0.04599 |  |
|  | GM16 | 39473 | 39450 | 59711 | 0.00120 |  |
|  | GM14 | 40471 | 40180 | 60810 | 0.01397 |  |
|  | GM12 | 40122 | 40450 | 61235 | -0.01569 |  |
|  | GM11 | 40687 | 39960 | 60472 | 0.03565 |  |
|  | GM9 | 41519 | 40590 | 61441 | 0.04467 |  |
|  | GM2b | 40105 | 39640 | 60004 | 0.02268 |  |
|  | GM1b | 40323 | 39830 | 60296 | 0.02397 |  |
| Schapendoes | SD843 | 40160 | 38700 | 58601 | 0.07327 | 0.141±0.035 |
|  | SD934b | 43596 | 40310 | 60995 | 0.15890 |  |
|  | SD808a | 43617 | 40640 | 61488 | 0.14280 |  |
|  | SD793a | 43642 | 40730 | 61652 | 0.13910 |  |
|  | SD789a | 42390 | 39720 | 60075 | 0.13130 |  |
|  | SD787a | 43464 | 40180 | 60796 | 0.15930 |  |
|  | SD742a | 43789 | 40860 | 61858 | 0.13950 |  |
|  | SD725a | 43007 | 40510 | 61315 | 0.12010 |  |
|  | SD674b | 43018 | 40220 | 60875 | 0.13560 |  |
|  | SD629b | 44116 | 40340 | 61042 | 0.18250 |  |
|  | SD604a | 44296 | 40090 | 60683 | 0.20440 |  |
|  | SD112b | 41590 | 39760 | 60197 | 0.08932 |  |
|  | SD111a | 42829 | 39730 | 60147 | 0.15170 |  |
| Berger des Pyrenées | BDP57 | 40223 | 40610 | 61459 | -0.01879 | 0.044±0.048 |
|  | BDP56b | 41748 | 40700 | 61598 | 0.05014 |  |
|  | BDP51 | 42270 | 40810 | 61774 | 0.06955 |  |
|  | BDP36 | 40242 | 39900 | 60360 | 0.01667 |  |
|  | BDP34 | 40850 | 40400 | 61134 | 0.02163 |  |
|  | BDP23b | 40287 | 40710 | 61616 | -0.02027 |  |
|  | BDP20a | 41628 | 40290 | 60965 | 0.06471 |  |
|  | BDP19a | 41298 | 40200 | 60819 | 0.05317 |  |
|  | BDP17a | 43400 | 41010 | 62089 | 0.11340 |  |
|  | BDP16b | 43541 | 40610 | 61448 | 0.14060 |  |
|  | BDP13 | 40849 | 40890 | 61898 | -0.00178 |  |
|  | BDP11a | 41905 | 40540 | 61337 | 0.06585 |  |
|  | BDP5 | 41391 | 41070 | 62176 | 0.01518 |  |

**Table S2. Inbreeding coefficient analysis for the genotyped dogs.** Inbreeding coefficient analysis for the genotyped dogs using PLINK with standard settings applied to the Array version 2 full filtered dataset (66,164 SNPs) according to the following criteria: Maximum 40% genotypes per locus missing; maximum 60% heterozygosity rate per locus; passing HWE-threshold of 0.05. From left to right: breed, individual ID (IID), number of observed homozygous loci [(O(hom)], number of expected homozygous loci [(E(hom)], number of non-missing genotypes used for the calculation [N(NM)], the inbreeding coefficient estimate (F) and the mean F for the corresponding breed ± SD.

| **breed** | **IID** | **NSEG** | **kb** | **KBAVG/dog ± SD [kb]** | **mean NSEG/breed**  **± SD** | **mean KBAVG/breed ± SD [kb]** |
| --- | --- | --- | --- | --- | --- | --- |
| Weimaraner | LW78 | 11 | 41313.5 | 3755.77±1098.35 | 10.92±4.29 | 4177.73±491.81 |
|  | LW63a | 11 | 48576 | 4416.00±1190.64 |  |  |
|  | LW58 | 6 | 18590.9 | 3098.48±992.93 |  |  |
|  | LW42 | 8 | 36327.7 | 4540.96±1469.41 |  |  |
|  | LW37 | 5 | 18815.2 | 3763.04±358.75 |  |  |
|  | LW34 | 15 | 64322.7 | 4288.18±1763.41 |  |  |
|  | LW33 | 8 | 33589.4 | 4198.68±1901.71 |  |  |
|  | LW31 | 8 | 34516.4 | 4314.55±749.56 |  |  |
|  | LW30 | 18 | 64952.2 | 3608.45±1066.58 |  |  |
|  | LW16 | 8 | 39491.7 | 4936.46±1493.43 |  |  |
|  | LW8 | 14 | 61612.1 | 4400.86±1462.28 |  |  |
|  | LW7 | 12 | 52815.3 | 4401.271388.05 |  |  |
|  | LW1 | 18 | 82579.6 | 4587.75±1668.63 |  |  |
| Large Munsterlander | GM31 | 10 | 45528.1 | 4552.81±1931.84 | 6.31±2.90 | 4069.70±744.68 |
|  | GM29 | 10 | 41187.8 | 4118.78±1032.86 |  |  |
|  | GM27 | 8 | 35496.6 | 4437.08±1572.57 |  |  |
|  | GM25 | 7 | 33856.7 | 4836.67±1632.92 |  |  |
|  | GM22 | 2 | 5827.49 | 2913.75±969.43 |  |  |
|  | GM21 | 6 | 25603.9 | 4267.31±1225.84 |  |  |
|  | GM16 | 6 | 28247.3 | 4707.89±1416.50 |  |  |
|  | GM14 | 5 | 18660.8 | 3732.16±1359.05 |  |  |
|  | GM12 | 3 | 13004.8 | 4334.92±1265.46 |  |  |
|  | GM11 | 7 | 30647.2 | 4378.17±1999.21 |  |  |
|  | GM9 | 9 | 36464.1 | 4051.56±2012.56 |  |  |
|  | GM2b | 1 | 2189.87 | 2189.87±0 |  |  |
|  | GM1b | 8 | 35080.6 | 4385.07±778.23 |  |  |
| Schapendoes | SD843 | 2 | 5757.37 | 2878.69±420.85 | 24.15±9.25 | 4475.81±560.95 |
|  | SD934b | 34 | 147675 | 4343.39±1630.13 |  |  |
|  | SD808a | 27 | 130636 | 4838.36±2278.63 |  |  |
|  | SD793a | 26 | 133505 | 5134.79±2571.23 |  |  |
|  | SD789a | 17 | 77172.2 | 4539.54±1778.36 |  |  |
|  | SD787a | 23 | 104213 | 4530.99±2505.44 |  |  |
|  | SD742a | 36 | 170902 | 4747.28±1996.25 |  |  |
|  | SD725a | 24 | 101498 | 4229.10±1385.95 |  |  |
|  | SD674b | 18 | 79426.1 | 4412.56±1661.34 |  |  |
|  | SD629b | 24 | 104650 | 4360.42±2305.91 |  |  |
|  | SD604a | 38 | 196190 | 5162.91±2289.69 |  |  |
|  | SD112b | 22 | 97576.6 | 4435.30±1831.65 |  |  |
|  | SD111a | 23 | 105160 | 4572.17±1858.09 |  |  |
| Berger des Pyrenées | BDP57 | 13 | 53045.3 | 4080.41±1584.06 | 14.69±6.90 | 4680.94±683.50 |
|  | BDP56b | 13 | 60070.2 | 4620.79±957.90 |  |  |
|  | BDP51 | 20 | 110348 | 5517.40±2346.51 |  |  |
|  | BDP36 | 11 | 42664.6 | 3878.60±1214.21 |  |  |
|  | BDP34 | 15 | 55079.7 | 3671.98±1261.12 |  |  |
|  | BDP23b | 7 | 25690.3 | 3670.05±818.09 |  |  |
|  | BDP20a | 13 | 71762.1 | 5520.16±2439.31 |  |  |
|  | BDP19a | 13 | 63165 | 4858.85±2024.89 |  |  |
|  | BDP17a | 31 | 160201 | 5167.78±2536.27 |  |  |
|  | BDP16b | 22 | 102897 | 4677.16±1761.58 |  |  |
|  | BDP13 | 6 | 31061.3 | 5176.89±1715.31 |  |  |
|  | BDP11a | 19 | 86325.4 | 4543.44±1976.58 |  |  |
|  | BDP5 | 8 | 43750.2 | 5468.77±3072.99 |  |  |

**Table S3. Runs of homozygosity using PLINK analysis.** Runs of homozygosity using PLINK analysis standard settings applied to the Array version 2 full filtered dataset (66,164 SNPs) according to the following criteria: Maximum 40% genotypes per locus missing; maximum 60% heterozygosity rate per locus; passing HWE-threshold of 0.05. From left to right: breed, individual ID (IID), number of homozygous segments per dog (NSEG), total homozygous distance spanned in kilobases per dog (kb), mean homozygous distance spanned per segment in kb per dog ± SD (KBAVG/dog ± SD), mean number of homozygous segments per breed ± SD (mean NSEG/breed ± SD), mean homozygous distance in kb spanned per segment per breed ± SD (KBAVG/breed ± SD [kb]).

| **comparison of groups** | **SNP** | **test** | **cases** | **controls** | **χ^2^** | **df** | **p-value** |
| --- | --- | --- | --- | --- | --- | --- | --- |
| pointing *vs.* herding dogs | rs23066192 | geno | 172/0/0 | 43/70/52 | 199.3 | 2 | 5.17*10^-44^ |
|  |  | trend | 344/0 | 156/174 | 167.7 | 1 | 2.32*10^-38^ |
|  |  | allelic | 344/0 | 156/174 | 244.5 | 1 | 4.10*10^-55^ |
|  |  | dom | 172/0 | 43/122 | 64.1 | 1 | 1.19*10^-15^ |
|  |  | rec | 172/0 | 113/52 | 199.3 | 1 | **2.91*10^-45^** |
|  | rs23041730 | geno | 168/0/0 | 49/28/88 | 181.2 | 2 | 4.40*10^-40^ |
|  |  | trend | 336/0 | 126/204 | 166.1 | 1 | 5.13*10^-38^ |
|  |  | allelic | 336/0 | 126/204 | 299.4 | 1 | 4.40*10^-67^ |
|  |  | dom | 168/0 | 49/116 | 121.8 | 1 | 2.58*10^-28^ |
|  |  | rec | 168/0 | 77/88 | 181.2 | 1 | **2.59*10^-41^** |
| pointing *vs*. other hunting dogs | rs23066192 | geno | 172/0/0 | 51/39/30 | 129.5 | 2 | 7.57*10^-29^ |
|  |  | trend | 344/0 | 141/99 | 112.0 | 1 | 3.58*10^-26^ |
|  |  | allelic | 344/0 | 141/99 | 170.9 | 1 | 4.79*10^-39^ |
|  |  | dom | 172/0 | 51/69 | 47.9 | 1 | 4.43*10^-12^ |
|  |  | rec | 172/0 | 30/90 | 129.5 | 1 | 5.27*10^-30^ |
|  | rs23041730 | geno | 168/0/0 | 52/25/42 | 123.4 | 2 | **1.60*10^-27^** |
|  |  | trend | 336/0 | 129/109 | 110.6 | 1 | 7.10*10^-26^ |
|  |  | allelic | 336/0 | 129/109 | 190.0 | 1 | 3.25*10^-43^ |
|  |  | dom | 168/0 | 52/67 | 69.5 | 1 | 7.80*10^-17^ |
|  |  | rec | 168/0 | 42/77 | 123.4 | 1 | **1.14*10^-28^** |
| pointing *vs*. other hunting and herding dogs | rs23066192 | geno | 172/0/0 | 94/109/82 | 198.0 | 2 | 9.92*10^-44^ |
|  |  | trend | 344/0 | 297/273 | 164.2 | 1 | 1.36*10^-37^ |
|  |  | allelic | 344/0 | 297/273 | 234.9 | 1 | 5.02*10^-53^ |
|  |  | dom | 172/0 | 94/191 | 60.3 | 1 | 8.11*10^-15^ |
|  |  | rec | 172/0 | 203/82 | 198.0 | 1 | **5.59*10^-45^** |
|  | rs23041730 | geno | 168/0/0 | 101/53/130 | 181.9 | 2 | 3.17*10^-40^ |
|  |  | trend | 336/0 | 255/313 | 162.7 | 1 | 2.95*10^-37^ |
|  |  | allelic | 340/0 | 255/313 | 285.9 | 1 | 3.85*10^-64^ |
|  |  | dom | 168/0 | 101/183 | 107.9 | 1 | 2.76*10^-25^ |
|  |  | rec | 168/0 | 154/130 | 181.9 | 1 | **1.87*10^-41^** |

**Table S4. Alternate / full model association tests using PLINK for the two candidate SNPs rs23066192 (*SETDB2* gene) and rs23041730 (*CYSLTR2* gene).** Pointing dogs (cases - n=172; 1 English Setter, 7 German Longhaired Pointing Dogs, 6 Gordon Setters, 5 Irish Setters, 75 Large Munsterlanders and 78 Weimaraner dogs) were compared to other hunting dogs including wolves (controls - n= 120; 23 Dachshunds, 2 Flat Coated Retrievers, 45 Glen of Imaal Terriers, 8 Golden Retrievers, 21 Labrador Retrievers, 18 German Wachtelhunds and 3 wolves), to herding dogs (controls - n= 165; 42 Berger des Pyrenées, 41 Giant Schnauzers, 14 Kuvasz and 68 Schapendoes) and to a combined group consisting of other hunting dogs and wolves and herding dogs. The analysis comprises Chi-Square (χ^2^) testing for genotypic (geno), Cochran-Armitage trend (trend), allelic, dominant (dom) and recessive (rec) models. p-values were automatically adjusted by PLINK v1.07 to the corresponding degrees of freedom (df). The initial hypothesis of a recessive inheritance model is strongly supported by the corresponding p values (bold) in comparison to the dominant model.

|  | | **haplotype frequencies** | | | | |
| --- | --- | --- | --- | --- | --- | --- |
| **SNP combina-tions** | **haplo-types** | **all dogs** | **pointing dogs** | **German Shorthaired**  **Pointing Dogs** | **other hunting dogs** | **herding dogs** |
| rs23041730 and rs23041728 | TT | 0.083 | 0.000 | 0.050 | 0.123 | 0.130 |
|  | TC | 0.297 | 0.000 | 0.675 | 0.349 | 0.470 |
|  | CT | 0.613 | 1.000 | 0.275 | 0.518 | 0.388 |
|  | CC | 0.007 | 0.000 | 0.000 | 0.010 | 0.012 |
|  | r^2^ | 0.663 | 1.000 | 0.788 | 0.563 | 0.545 |
|  | D' | 0.965 | 1.000 | 1.000 | 0.949 | 0.938 |
|  |  |  |  |  |  |  |
| rs23066192 and rs23041730 | TT | 0.291 | 0.000 | 0.675 | 0.363 | 0.485 |
|  | TC | 0.027 | 0.000 | 0.000 | 0.053 | 0.042 |
|  | CT | 0.072 | 0.000 | 0.050 | 0.095 | 0.133 |
|  | CC | 0.611 | 1.000 | 0.275 | 0.489 | 0.34 |
|  | r^2^ | 0.616 | 1.000 | 0.788 | 0.495 | 0.432 |
|  | D' | 0.867 | 1.000 | 1.000 | 0.766 | 0.792 |

**Table S5. Haplotype frequencies for SNPs combinations rs23041730 and rs23041728 in the *CYSLTR2* gene and rs23066192 and rs23041730 in the *SETDB2* and *CYSLTR2* genes.** High linkage disequilibrium (LD) is present as indicated by the r² and D’ values for the entire dog cohort as well as corresponding subgroups, showing highest LD for the pointing dogs. All dogs (n=477, pointing dogs, German Shorthaired Pointing Dogs, other hunting dogs including wolves and herding dogs), pointing dogs (n=172; 1 English Setter, 7 German Longhaired Pointing Dogs, 6 Gordon Setters, 5 Irish Setters, 75 Large Munsterlanders and 78 Weimaraner dogs), 20 German Shorthaired Pointing Dogs, other hunting dogs including wolves (n= 120; 23 Dachshunds, 2 Flat Coated Retrievers, 45 Glen of Imaal Terriers, 8 Golden Retrievers, 21 Labrador Retrievers, 18 German Wachtelhunds and 3 wolves) and herding dogs (n= 165; 42 Berger des Pyrenées, 41 Giant Schnauzers, 14 Kuvasz and 68 Schapendoes).

| **A)** | | | | | | **Pointing *vs.* herding dogs** | | | | |  | | | | |
| --- | --- | --- | --- | --- | --- | --- | --- | --- | --- | --- | --- | --- | --- | --- | --- |
|  |  | **Array version 2 platinum set** | | | |  | **Array version 2 full set PLINK filtered** | | | |  | **Array version 2 full set PLINK + 60% het/locus filtered** | | | |
| **chr** | **bp range** | **LW** | **GM** | **SD** | **BDP** | **bp range** | **LW** | **GM** | **SD** | **BDP** | **bp range** | **LW** | **GM** | **SD** | **BDP** |
| 8 | 4521970 - 6494289 | 1.0 |  |  |  | - |  |  |  |  | - |  |  |  |  |
| 11 | 27861509 - 29734366 |  | 0.8 |  |  | 28143152 - 29837082 |  | 0.9 |  |  | 29157734 - 29734366 | 0.8 | 0.8 | 0.8 |  |
| 20 | 24150120 - 25781768 | 0.9 |  |  |  | 24804061 - 24969549 | 0.8 |  |  |  | 23697631 - 25427246 | 0.8 |  |  |  |
| 22 | 3067105 - 6598327 | **0.9** | **0.9** |  |  | 5093150 - 6163861 | **0.9** | **0.9** |  |  | 5052718 - 6163861 | **1.0** | **1.0** |  |  |
| 24 | - |  |  |  |  | 3783384 - 3938709 | 0.8 | 0.8 | 0.8 |  | - |  |  |  |  |
| 30 | - |  |  |  |  | 3870300 - 5345381 | 1.0 |  | 1.0 |  | 3870300 - 5127075 | 0.9 |  | 0.9 |  |
| 34 | - |  |  |  |  | 3765052 - 4820892 |  |  | 0.8 |  | - |  |  |  |  |
| **B)** | | | | | | **Herding *vs.* pointing dogs** | | | | |  | | | | |
|  |  | **Array version 2 platinum set** | | | |  | **Array version 2 full set PLINK filtered** | | | |  | **Array version 2 full set PLINK + 60% het/locus filtered** | | | |
| **chr** | **bp range** | **LW** | **GM** | **SD** | **BDP** | **bp range** | **LW** | **GM** | **SD** | **BDP** | **bp range** | **LW** | **GM** | **SD** | **BDP** |
| 10 | 7927964 - 11815748 |  |  |  | 0.9 |  |  |  |  |  | - |  |  |  |  |
| 13 | 10182231 - 12964124 |  |  | **1.0** | **1.0** | 10324590 - 12964124 |  |  | **1.0** | **1.0** | 10324590 - 12964124 |  |  | **1.0** | **1.0** |
| 13 | 37155827 - 39172713 |  |  | 0.9 |  | - |  |  |  |  | - |  |  |  |  |
| 16 | 38644141 - 38982035 |  |  | 0.8 |  | - |  |  |  |  | - |  |  |  |  |
| 30 | 3870300 - 4322901 | 0.8 |  | 0.8 |  | 3926674 - 4799522 | 0.8 |  | 0.8 |  | 3926674 - 4799522 | 0.8 |  | 0.8 |  |

**Table S6. Homozygous genomic regions identified by Homozygosity Mapper**. Homozygous genomic regions identified by Homozygosity Mapper exceeding the minimum threshold of 0.8, using standard settings for the given number of genotyped SNPs as well as three different input files as described in the section “Array Based Genotyping” in the main article. The number of analyzed SNPs per file was lower than the number of input SNPs (file 1 - 49,024 of 49,663 SNPs; file 2 - 65,740 of 66,915 SNPs ; file 3 - 65,132 of 66,164 SNPs) due to missing probe IDs provided by the manufacturer. Results are presented for each breed. Inspection and evaluation of the identified homozygous regions revealed breed specific homozygosity. Panel A) shows the results for the comparison of hunting (cases) *vs.* herding (controls) dogs. Panel B) shows the results for the comparison of herding (cases) *vs.* hunting (controls) dogs. From left to right – identified chromosome (chr), the chromosomal position given in base pair (bp) range and the individual breeds: Weimaraner (LW), Large Munsterlander (GM), Schapendoes (SD) and Berger des Pyrenées (BDP). Candidate regions were defined as regions showing recurrent hits exceeding the 0.8 threshold in Homozygosity Mapper for the three analyzed file sets in hunting or herding dogs as indicated in bold.


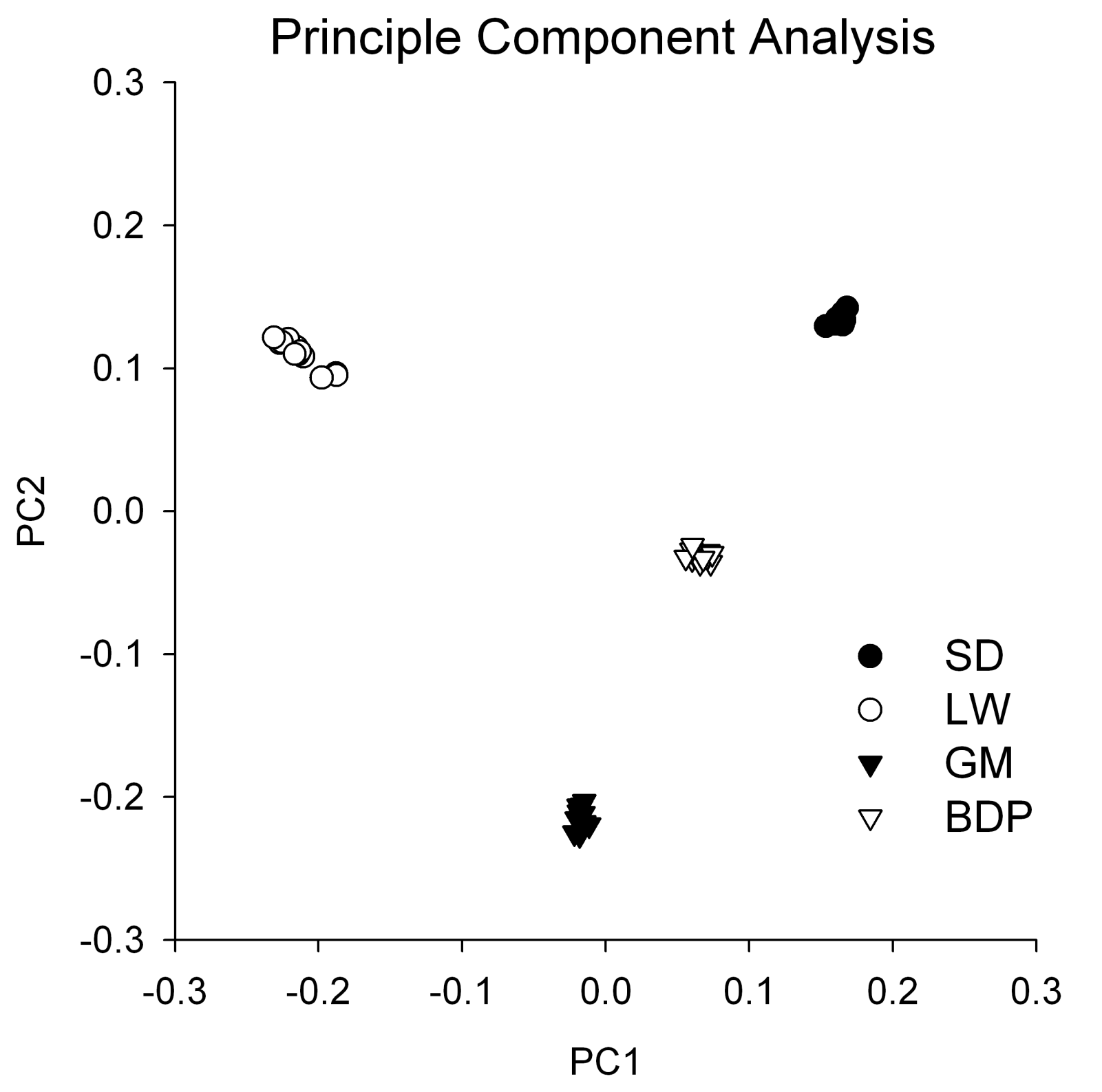


**Figure S1. Principle Component Analysis (PCA).** Principle Component Analysis (PCA) results from SNPRelate for the Array version 2 full filtered dataset (66,164 SNPs) according to the following criteria: Maximum 40% genotypes per locus missing; maximum 60% heterozygoitys rate per locus; passing HWE-threshold of 0.05. Plotting the eigenvectors PC1 and PC2 for each dog, four clusters are distinguishable. The individual IDs identify each cluster to represent a distinct breed. Weimaraner (LW) and Large Munsterlander (GM), Schapendoes (SD) and Berger des Pyrenées (BDP).


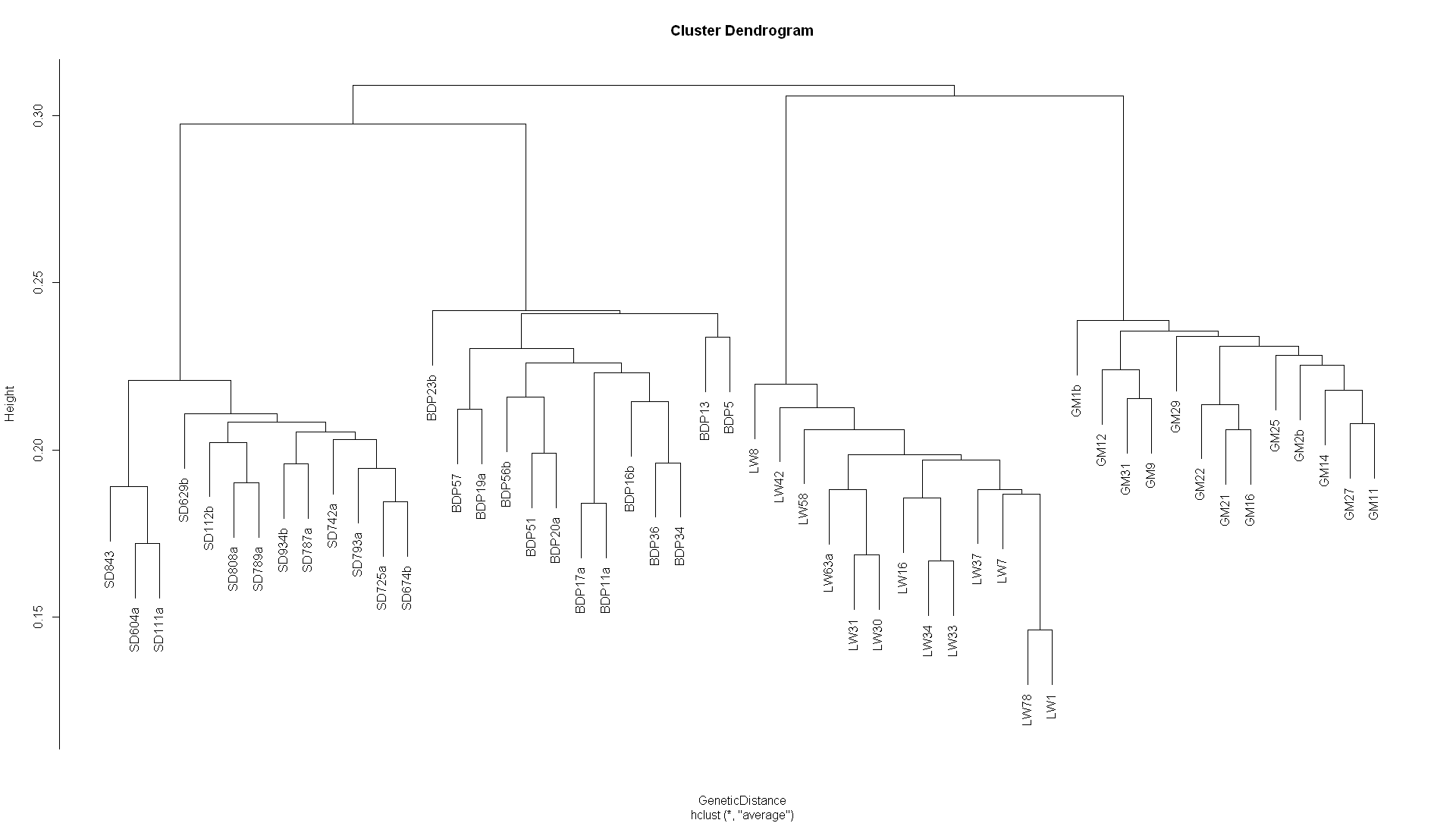


**Figure S2. Cluster dendrogram.** Cluster dendrogram from SNPRelate applied to the Array version 2 full filtered dataset (66,164 SNPs) according to the following criteria: Maximum 40% genotypes per locus missing; maximum 60% heterozygosity rate per locus; passing HWE-threshold of 0.05. The dendrogram confirms the initial grouping of herding and hunting dogs as indicated by the upper two clades. Grouping the Weimaraner (LW) and Large Munsterlander (GM) dogs reveals higher similarity in comparison to the other group of Schapendoes (SD) and Berger des Pyrenées (BDP). The height of the vertical lines indicates the degree of similarity with longer distances indicating less relatedness. The results are in line with the data from PCA (see Figure S1) identifying four distinct breeds.


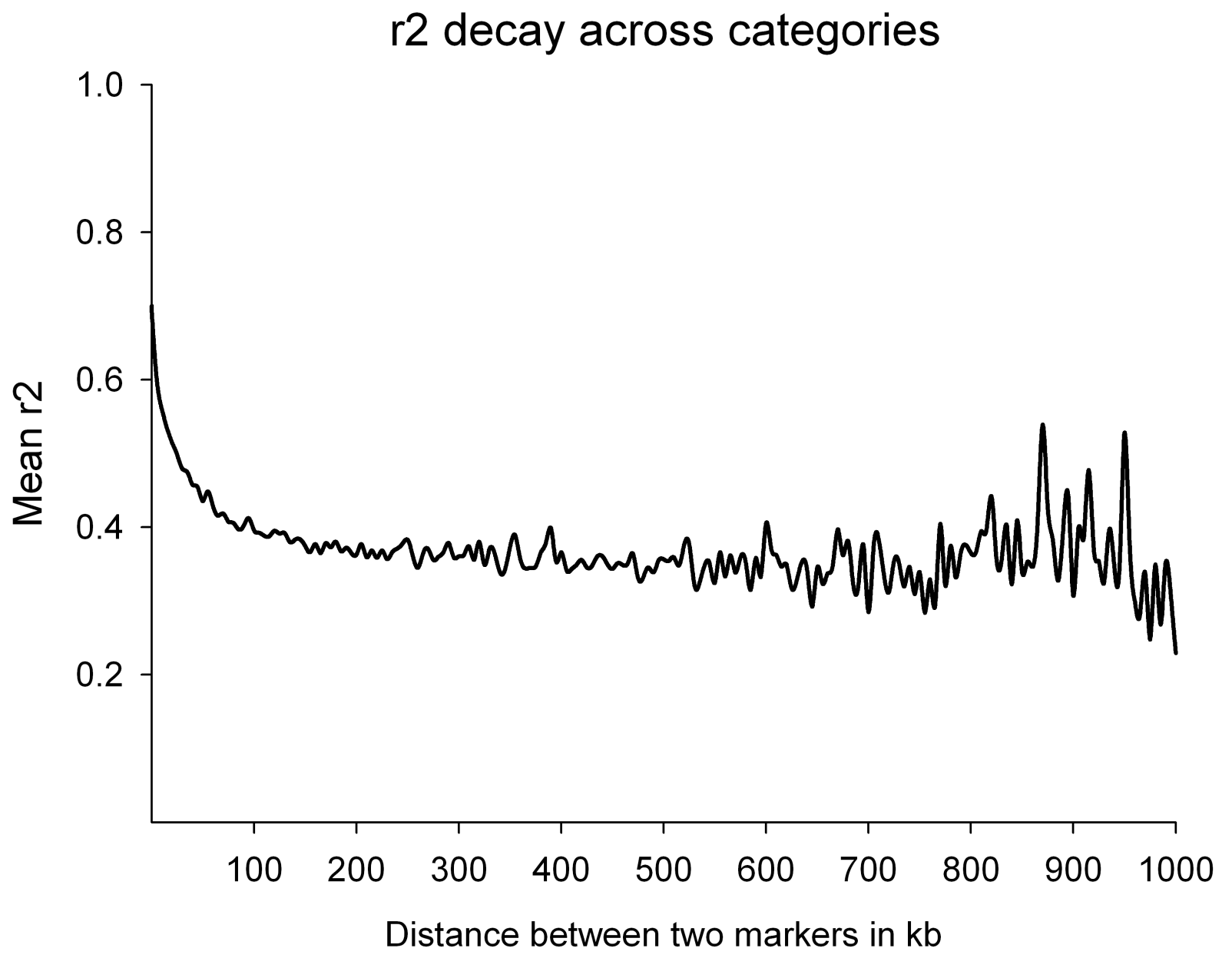


**Figure S3. Average r^2^ decay plot.** The average r^2^ decay plot was generated for across breed categories. As shown before (Lindblad-Toh et al. 2005), the overall dog linkage disequilibrium decreases rapidly reaching the baseline level at approximately 200-300 kb.


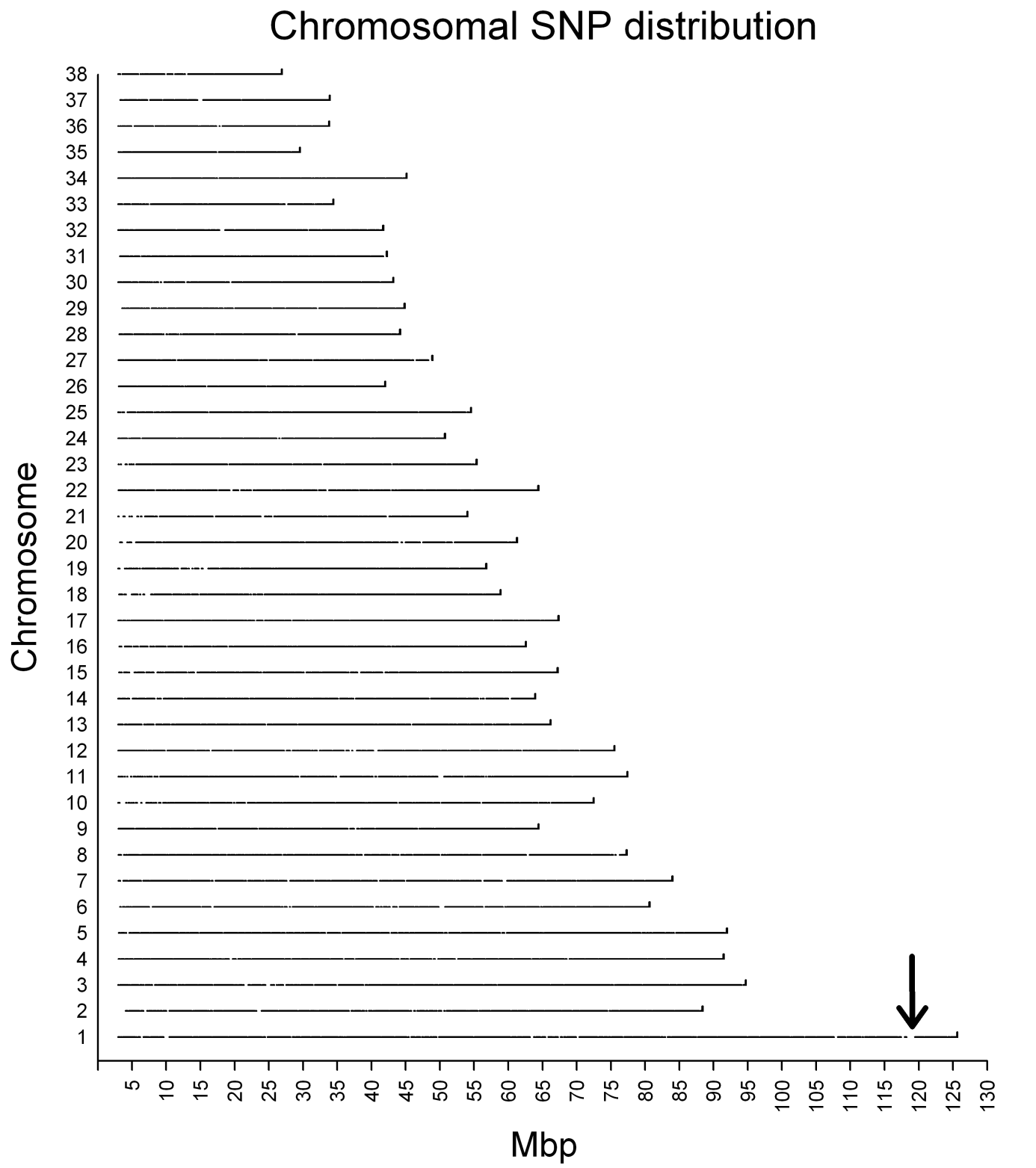


**Figure S4. Chromosomal SNP distribution.** SNP distribution for each chromosome for the Array version 2 full filtered dataset (66,164 SNPs) according to the following criteria: Maximum 40% genotypes per locus missing; maximum 60% heterozygosity rate per locus; passing HWE-threshold of 0.05. SNPs are indicated by dots, appearing as dotted line. Chromosomal regions not covered by SNPs can be identified by gaps as exemplarily shown by the arrow. The small vertical bars at the right end of each chromosome indicate the physical ends; information as extracted from the UCSC may 2005 dog (*Canis familiaris*) whole genome shotgun (WGS) assembly v2.0.


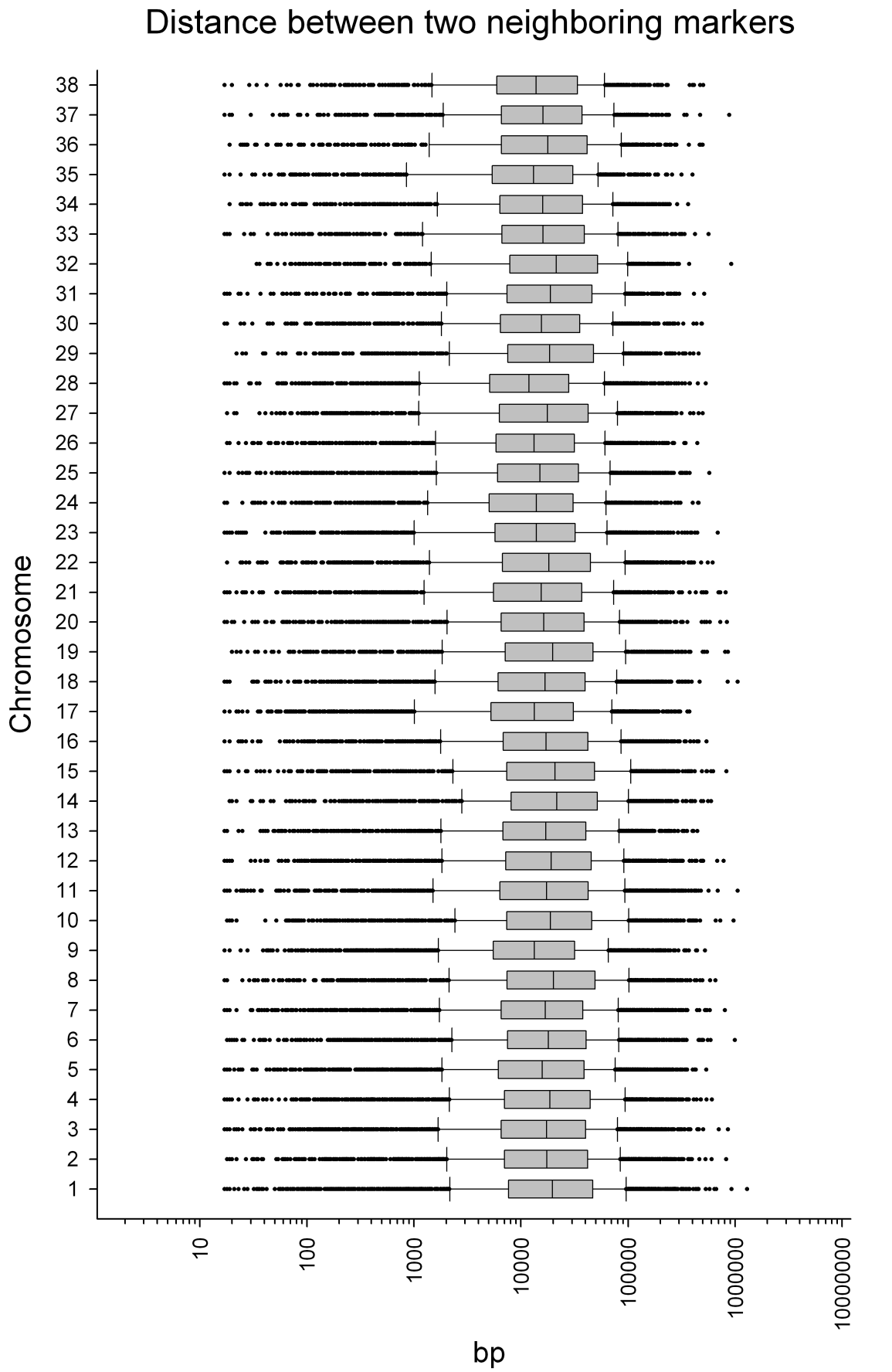


**Figure S5. Median marker distances.** Median distances are plotted between two neighbouring markers as indicated by vertical lines in boxes for the Array version 2 full filtered dataset (66,164 SNPs) according to the following criteria: Maximum 40% genotypes per locus missing; maximum 60% heterozygosity rate per locus; passing HWE-threshold of 0.05. Boxes indicate 25^th^ and 75^th^ percentiles, error bars indicate 90^th^ and 10^th^ percentiles, and dots indicate outliers.
